# The Copper-microRNA Pathway Is Integrated with Developmental and Environmental Stress Responses in *Arabidopsis thaliana*

**DOI:** 10.3390/ijms22179547

**Published:** 2021-09-02

**Authors:** Ana Perea-García, Amparo Andrés-Bordería, Peter Huijser, Lola Peñarrubia

**Affiliations:** 1 Departament de Bioquímica i Biologia Molecular and Institut Universitari de Biotecnologia i Biomedicina (BIOTECMED), Universitat de València, 46100 Burjassot, Valencia, Spain; ana.perea@uv.es (A.P.-G.); dandres@cipf.es (A.A.-B.); 2 Department of Comparative Development and Genetics, Max Planck Institute for Plant Breeding Research, Carl-von-Linné-Weg 10, 50829 Cologne, Germany; huijser@mpipz.mpg.de

**Keywords:** *Arabidopsis thaliana*, copper homeostasis, copper-microRNAs, development, SPL, stress

## Abstract

As an essential nutrient, copper (Cu) scarcity causes a decrease in agricultural production. Cu deficiency responses include the induction of several microRNAs, known as Cu-miRNAs, which are responsible for degrading mRNAs from abundant and dispensable cuproproteins to economize copper when scarce. Cu-miRNAs, such as miR398 and miR408 are conserved, as well as the signal transduction pathway to induce them under Cu deficiency. The *Arabidopsis thaliana* SQUAMOSA-PROMOTER BINDING PROTEIN-LIKE (SPL) family member SPL7 binds to the *cis*-regulatory motifs present in the promoter regions of genes expressed under Cu deficiency, including *Cu-miRNAs*. The expression of several other SPL transcription factor family members is regulated by *miR156*. This regulatory *miR156-SPL* module plays a crucial role in developmental phase transitions while integrating internal and external cues. Here, we show that Cu deficiency also affects *miR156* expression and that *SPL3* overexpressing plants, resistant to *miR156* regulation, show a severe decrease in SPL7-mediated Cu deficiency responses. These include the expression of *Cu-miRNAs* and their targets and is probably due to competition between SPL7 and *miR156*-regulated SPL3 in binding to *cis*-regulatory elements in *Cu-miRNA* promoters. Thus, the conserved SPL7-mediated Cu-miRNA pathway could generally be affected by the *miR156-SPL* module, thereby underscoring the integration of the Cu-miRNA pathway with developmental and environmental stress responses in *Arabidopsis thaliana.*

## 1. Introduction

The deficiency of essential nutrients, such as copper (Cu), causes a decrease in agricultural productivity and crop performance. Copper participates as a cofactor in multiple plant physiological processes and the main symptoms produced by its deficiency affect young leaves and reproductive organs, reducing plant fertility and grain/seed yield [1]. While copper redox properties are involved in its essential functions, they can also produce hazardous reactive oxygen species (ROS). To accomplish copper requirements while avoiding toxicity requires a fine modulation of homeostatic networks for copper distribution [2,3,4,5]. In *Chlamydomonas reinhardtii*, Cu deficiency responses were shown to be driven by the master transcription factor Copper Response Regulator 1 (CRR1) [6,7]. Remarkably, given the evolutionary distance, its closest homologue in *Arabidopsis*, SPL7, turned out to be a master regulator of Cu deficiency responses too [8,9]. Both, CRR1 and SPL7 are members of the SQUAMOSA-PROMOTER BINDING PROTEIN-LIKE (SPL) family of transcription factors (TFs).

In *Arabidopsis thaliana,* the SPL TFs are mainly involved in the control of developmental phase transitions, and associated with reproduction in particular [10]. The SPL family members share a conserved DNA binding domain, denoted as SBP-domain, and have been exclusively found in the green plant lineage [11]. The 16 members of the *A. thaliana* SPL family can be grouped in two subfamilies based on their sizes and similarities [12,13]. Large SPLs include SPL1, SPL7, SPL12, SPL14 and SPL16, whereas the remaining 11 SPLs are grouped as small SPLs [13]. With the exception of *SPL8*, the small *SPLs* also share a functional miRNA response element (MRE) downstream of the conserved SBP box that is complementary to *miR156* and the closely related *miR157* [14,15,16,17]. Hereafter, these *SPLs* will be denoted as *MRE-SPLs* since they are targeted post-transcriptionally by *miR156* and *miR157*. Although *miR156* and *miR157* are both abundantly expressed before and less during the vegetative and reproductive phase transitions [15], genetic analysis showed that miR156 plays a more important role than miR157 in the timing of these phase transitions [18].

Whereas the floral transition involves the reduction of *miR156/157* expression, another miRNA, *miR172*, becomes induced and promotes flowering through the posttranscriptional regulation of different AP2-like TFs [19,20]. Since the levels of *miR156* decline as the plant ages [16,21,22], a subsequent gradual increase in the expression of *miR156*-targeted *MRE-SPLs* is observed. This process constitutes a regulatory module (*miR156-SPL*) for proper plant life cycle progression [16,22,23,24,25,26,27,28]. Constitutive overexpression of *miR156* results in the delay of the juvenile-to-adult phase transition and mutations that render its *MRE-SPL* targets insensitive to *miR156* have the opposite effect. For instance, the absence of a functional MRE in a constitutive promoter-driven *SPL3* transgene has allowed the *miR156/157*-independent expression of the SPL3 protein in transgenic plants. As a consequence, these plants showed a shortened juvenile phase and obtained an early flowering phenotype [16,17]. This fact suggests that vegetative phase change in higher plants is primarily controlled by the *miR156-SPL* module. Furthermore, the expression of several *miR156* genes has been found to become induced under various stress conditions, and the decrease in *miR156* expression when conditions normalized has been associated with preparing for flowering, denoting that the *miR156-SPL* module coordinates development while integrating multi-stress responses [29,30].

Transcription factor families generally expand with increasing organism complexity, consistent with a need for refined control of specific subsets of target genes. The DNA-binding recognition motif for SBP-domain TFs contains a core motif of only four nucleotides, GTAC [31,32]. The large number of members recognizing this short and highly abundant motif in the genome raises the possibility of competitive binding and thereby interfering with specificity in target gene regulation [33]. It is conceivable that several SPLs overlap in their biological functions and in their ability to compensate for the loss of other SPL functions [13,16,27]. Whether or not different SPL members may compete for binding to GTAC containing response elements in target promoters remains unsolved. Outside the conserved SBP domain, the 16 proteins of the SPL family show limited amino acid conservation, probably reflecting the specific roles attained by the different SPL proteins [27]. Among the large SPLs, the specific and unique role of SPL7 in Cu deficiency responses seems unquestioned. Function of the paralogous *SPL1* and *SPL12* genes could not be linked to the copper response [34], but rather seem to be implicated in thermotolerance [35], whereas *spl14* mutants showed altered plant architecture [36].

The GTAC motifs bound by CRR1/SPL7 to regulate genes in response to Cu deficiency are known as Copper Response Elements (CuREs) [7,37,38]. Cu deficiency responses include the induction of several microRNAs (denoted as *Cu-miRNAs*) that target cuproproteins. These *Cu-miRNAs* consist of *miR397*, *miR398*, *miR408* and *miR857* [37,39,40]. *miR398* is responsible for degrading the mRNAs of genes (*CSD1*, *CSD2*) coding for Cu and zinc (Zn) superoxide dismutase (Cu/ZnSOD), the Cu chaperone CCS and for a subunit of the mitochondrial cytochrome *c* oxidase, COX5b-*1* [41,42,43]. Thus, one of the SPL7 functions in order to reserve copper for more essential processes is to replace Cu-ZnSOD with iron superoxide dismutase (FeSOD), encoded by *FSD1,* [8,37,44]. *miR398* is one of the most reported miRNAs to participate in abiotic and biotic stress regulatory networks. Copper, phosphate and water deficiencies, oxidative, salt and abscisic acid stresses, UV light and plant pathogens are just a few examples [45]. Accordingly, putative stress-responsive *cis*-elements were found in *miR398* promoters [46]. Furthermore, *CSD2* mRNA levels were not correlated with *miR398* levels under certain stress conditions, suggesting the existence of *miR398*-dependent and independent regulatory mechanisms for *CSD2* expression [46]. Due to the role of SODs as ROS scavengers, an important role of miR398 in oxidative stress resistance and plant survival has been postulated [47,48]. *miR398* expression, as well as its targets, suffer a diurnal oscillation at the transcriptional level [49]. These data indicate that the spatiotemporal patterns of *Cu-miRNA* expression may have an important role in the response to nutrient deficiency and add another layer of complexity to the study of their function.

*miR408* targets transcripts of genes encoding abundant cuproproteins that belong to the phytocyanin family, such as plantacyanin, and several laccases involved in the oxidative polymerization of lignin [50,51,52,53]. The *miR408* transcript levels respond to different abiotic stresses and elevated *miR408* levels contribute to improved tolerance to salinity, cold and oxidative stress, but enhanced sensitivity to drought, osmotic stress and Fe deficiency [53,54]. Constitutive expression of *miR408* results in enhanced vegetative growth, which led to suggestions of role as a modulator of development [55,56,57]. *miR408* is among the most conserved miRNA families in land plants, while *miR397* and *miR398* are conserved in all angiosperms [58,59,60].

It is believed that *Cu-miRNAs* eliminate the expression of particular cuproproteins and make the scarce copper available to essential cuproproteins of the organelle electron transport chains, such as plastocyanin and cytochrome *c* oxidase [61]. However, little is known about how the function of components upstream in the *Cu-miRNA* pathway, such as SPL7, is modulated to fine-tune this pathway according to developmental or stress signaling. The MRE-SPLs do not share conserved functional domains with SPL7 outside the DNA-binding domain which includes a nuclear localization signal [31]. However, both MRE-SPLs and SPL7 do recognize the same GTAC core element. Therefore, when co-expressed, some degree of target-binding competition cannot be ruled out with a likely negative impact on SPL7 function. A conceivable way to reinforce SPL7-mediated Cu deficiency responses could thus be the expression attenuation of other putative competing MRE-SPLs, e.g., through the expression of *miR156/157*.

In this study, we addressed two questions. First, is there any evidence for the role of the *miR156-SPL* module in the regulation of the Cu deficiency responses during plant development? Second, is the Cu-miRNA pathway globally or specifically influenced by developmental clues and by other abiotic stresses?

## 2. Results

### 2.1. miR156 and Its Targets Expression under Different Copper Content

**Figure 1:**
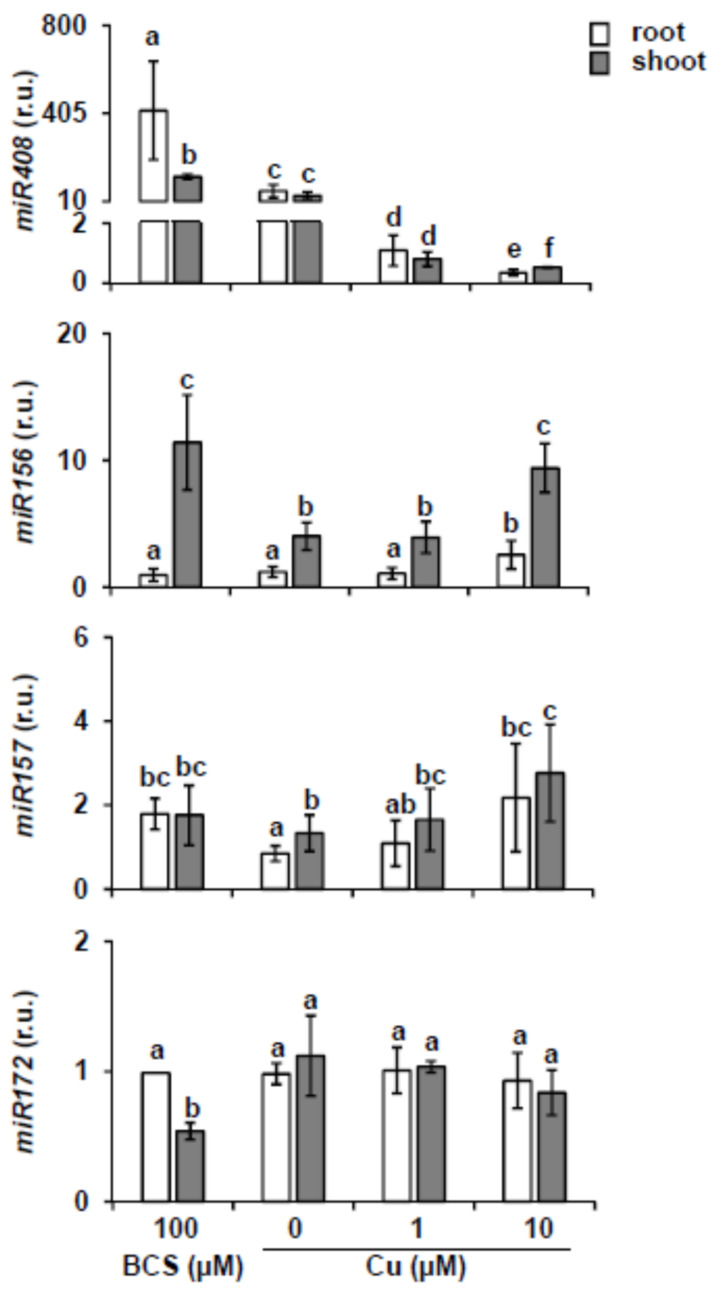
*miR408*, *miR156*, *miR157* and *miR172* expression levels at different copper concentrations. Relative expression of *miR408*, *miR156, miR157* and *miR172* genes in roots (white bars) and shoots (gray bars) from 7-day old WT seedlings grown under severe Cu deficiency (MS 0 μM Cu + 100 μM BCS), Cu deficiency (MS 0 μM Cu), Cu sufficiency (MS + 1 μM Cu, control) and Cu excess (MS + 10 μM Cu). Samples were collected at zeitgeber time 12 h. After miRNA extraction, specific primers were used for stem-loop RT-qPCR and expressed as relative units (r.u.). *18S* rRNA was used as internal control and the levels in the WT root sample grown under Cu sufficiency are arbitrarily set at one for comparison. The bars represent the mean ± SD of three biological replicates. Means with a different letter are significantly different (*p* < 0.05).

In *Arabidopsis*, *miR156* and *miR157* isoforms are encoded by ten (*miR156a-j*) and four loci (*miR157a-d*), respectively (Supplementary Table S1). With the aim to test whether *miR156* and *miR157* expression is regulated by the presence of copper in the media, we analyzed by stem-loop RT-qPCR *miR156* and *miR157* mature levels. To this end, roots and shoots from 7-day-old wild-type (WT) seedlings were separately collected after growing in different copper concentrations. To that end, WT seeds were grown on MS medium (0 Cu) and MS supplemented with 100 μM BCS (100 BCS), 1 μM Cu (1 Cu) or 10 μM Cu (10 Cu). These concentrations range from severe Cu deficiency (100 BCS), deficiency (0 Cu), sufficiency (1 Cu) to slight Cu excess (10 Cu). In order to confirm Cu deficiency, increase in the Cu-miRNA *miR408* levels was monitored as control. As shown in Figure 1, *miR408* expression increased both in roots and shoots as copper decreased in the media, with higher expression levels in roots under severe Cu deficiency. The analysis of the *miR156* transcript levels shows that it is higher in shoots, where it is induced under both excess and severe deficiency of copper with respect to Cu sufficiency (Figure 1). The results for *miR157* expression are similar to those obtained for *miR156* with slightly increased expression, but not significant, under both very low and high copper levels compared to control conditions. The main difference with *miR156* is that the expression levels in roots and shoots were similar for *miR157* (Figure 1). Furthermore, it has been described that *miR172* is downstream regulated by *miR156* [10,22], so we also analyzed in the same conditions the *miR172* expression. Accordingly, the pattern of *miR172* expression was the opposite of the *miR156* under severe Cu deficiency, showing slightly reduced expression (Figure 1).

In order to know if there is a *miR156* and *miR157* transcriptional regulation depending on the copper nutritional status, we used transgenic plants where different *miR156* (*a*, *b* and *d*) and *miR157* (*a*–*c*) promoters are fused to the reporter gene β-glucuronidase (GUS) (Figure S1) [13]. GUS activity directed by the *miR156a*, *miR156d* and *miR156h* promoters was detected in 7-day-old seedlings mainly in shoots under Cu deficiency (Figure S1). In the case of GUS activity for *miR157a*, *miR157b* and *miR157c*, it was detected in the cotyledon’s vascular bundles under Cu deficiency. *miR156* and *miR157* GUS activity is also observed in sufficiency and excess of copper (results not shown). Furthermore, analysis of promoter regions up to 2 kb upstream of the *miR156/157* pre-miRNA coding sequences indicates the presence of putative Cu deficiency *cis*-regulatory elements (GTAC boxes) in all of them (Supplementary Table S1).

**Figure 2:**
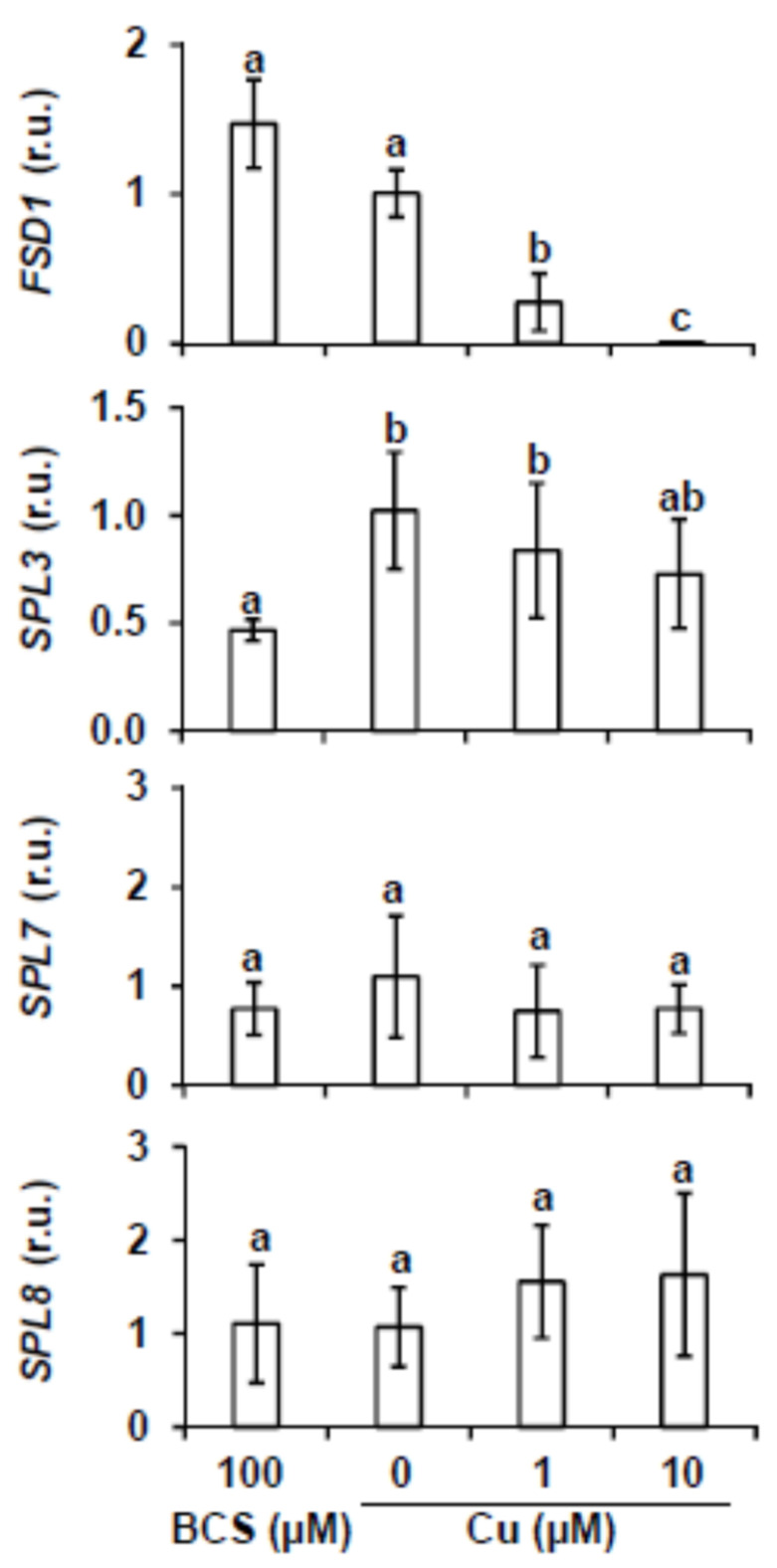
*FSD1*, *SPL3*, *SPL7* and *SPL8* expression at different copper concentrations. Relative expression of *SPL3*, *SPL7* and *SPL8* genes from 7-day old WT seedlings grown under severe Cu deficiency (MS 0 μM Cu + 100 μM BCS), Cu deficiency (MS 0 μM Cu), Cu sufficiency (MS + 1 μM Cu, control) and Cu excess (MS + 10 μM Cu). After total RNA extraction, specific primers were used for RT-qPCR and expressed as relative units (r.u.). *UBQ10* gene expression was used as internal control and levels in the WT sample grown under Cu sufficiency are arbitrarily set at one for comparison. The bars represent the mean ± SD of three biological replicates. Means with a different letter are significantly different (*p* < 0.05).

The miR156 and miR157 regulate the expression of *MRE-SPLs* at a posttranscriptional level, among these is *SPL3* [13,14]. Other *SPLs*, such as *SPL7* and *SPL8*, are not targets of *miR156/157*. In order to check the expression of *SPL* members, targets and non-targets of *miR156*, in response to copper availability, 7-day-old WT seedlings were grown on MS medium (0 μM Cu) and MS supplemented with 100 μM BCS (100 BCS), 1 μM Cu (1 Cu) or 10 μM Cu (10 Cu) and the transcript levels were analyzed by RT-qPCR (Figure 2). Up-regulation under Cu deficiency of *FSD1* expression, used as a control, confirmed proper Cu deficiency responsiveness of the analyzed samples (Figure 2). According to the increased *miR156/157* expression under severe Cu deficiency, *SPL3* was slightly down-regulated when BCS was added in the medium or when copper is in excess (Figure 2), whereas the relative expression of *SPL7* and *SPL8* genes was not significantly affected by copper treatment (Figure 2).

**Figure 3:**
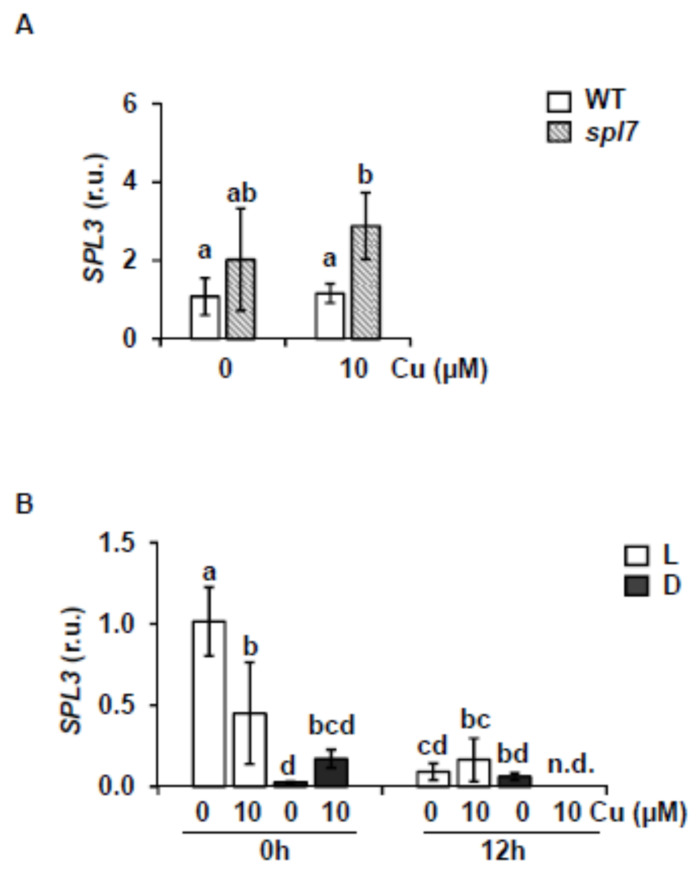
*SPL3* expression under different metal conditions. (**A**) *SPL3* relative expression in 7-day-old WT (white bars) and *spl7* (striped bars) seedlings grown under). (**B**) *SPL3* relative expression in 7-day-old WT seedlings grown under Cu deficiency (MS 0 μM Cu) and Cu excess (MS with 10 μM Cu) and neutral photoperiod (12L/12D; white bars) or etiolated plants (24D; gray bars). Samples were collected at zeitgeber time 0 and 12 h. After total RNA extraction, specific primers were used for RT-qPCR and expressed as relative units (r.u.). *UBQ10* gene was used as internal control and the WT sample grown under Cu deficiency at zeitgeber time 0 h of light is arbitrarily set at one for comparison. The bars represent the mean ± SD of three biological replicates. Means with a different letter are significantly different (*p* < 0.05). n.d.: not detected.

In order to know whether the *SPL3* expression under Cu deficiency is dependent on *SPL7* through the effect on *miR156*, we examined *SPL3* expression in 7-day-old WT and *spl7* mutant seedlings grown under Cu deficiency (MS) and Cu-excess (MS with 10 μM Cu). Results show that an increased *SPL3* expression is observed in *spl7* mutant seedlings compared to the WT under Cu-excess. However, *SPL3* expression under slight Cu deficiency was not significantly different (Figure 3A). Accordingly, *SPL3* expression under Cu-sufficiency was similar in WT and *spl7* seedlings, while it was significantly increased in the *spl7* mutant under severe Cu deficiency (data not shown). Moreover, since it was mostly expressed in flowers, *SPL3* transcript levels were also checked in this tissue (Figure S2A). Surprisingly, *SPL3* expression in flowers was reduced in the *spl7* mutant (Figure S2A). Nevertheless, since at least part of the *miR156*-dependent regulation is the result of translational inhibition (Gandikota et al., 2007), protein levels should be also checked. Instead, the expression of two of the SPL3 targets, *APETALA 1* (*AP1*) and *FRUITFULL* (*FUL*), was analyzed and accordingly reduced in flowers (Figure S2B,C). These results pointed to a complex regulation of *SPL3* depending on Cu status along development.

The presence of GTAC boxes in the *SPL3* promoter (data not shown) and the putative connection between copper homeostasis and the circadian clock [62] prompted us to further pursue the regulation of *SPL3* by copper in seedlings. To check *SPL3* expression under light/dark conditions (12 h light and temperature cycles; L) and continuous darkness (etiolated seedlings with 12 h temperature cycles; D), WT seedlings were grown for 7 d in Cu-deficient and Cu-excess media, samples were collected at zeitgeber time 0 and 12 h and gene expression was analyzed by RT-qPCR (Figure 3B). *SPL3* transcription shows a differential expression pattern between 0 and 12 h, being higher at 0 than at 12 h. However, there is a slight *SPL3* induction under slight Cu deficiency at 0 h in neutral photoperiod (12 h of Light and Dark, 23 °C Hot 16 °C Cold; LDHC) (Figure 3B). In etiolated plants, *SPL3* gene expression is attenuated regardless of the conditions (Figure 3B). These results point to a complex *SPL3* expression pattern probably depending on the light, circadian rhythms and copper content, maybe acting throughout interconnected pathways.

### 2.2. Phenotype of SPL3 Overexpressing Plants under Different Copper Status

**Figure 4:**
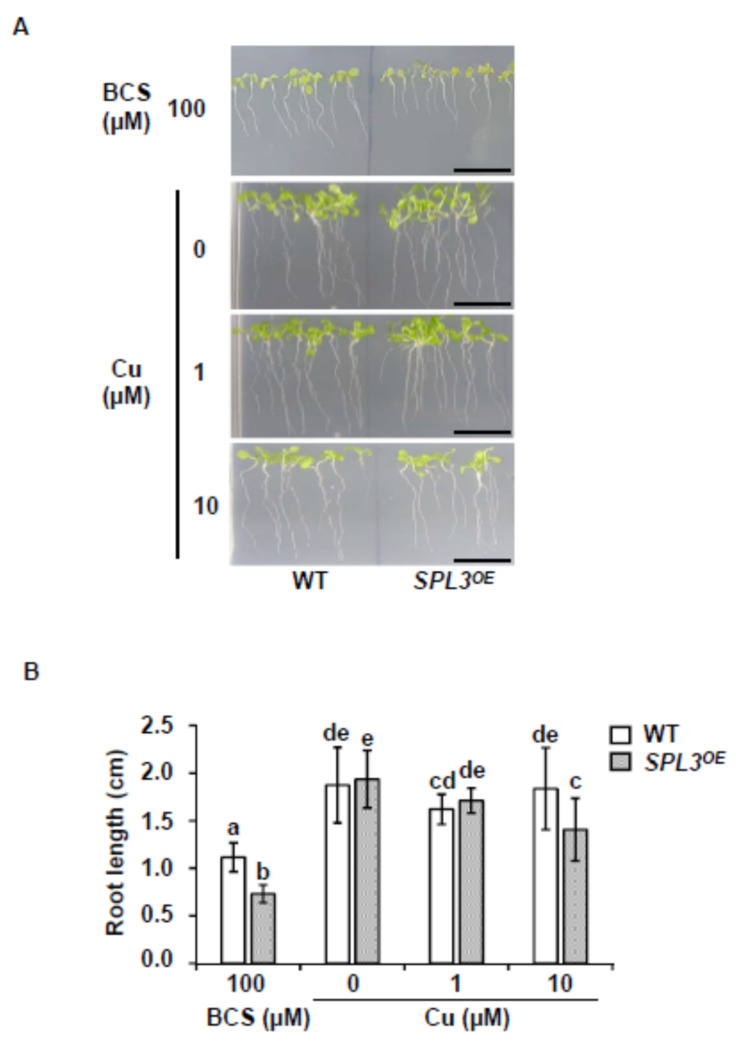
Phenotype of the *SPL3^OE^* seedlings at different copper concentrations. (**A**) Photographs of 7-day-old WT and *SPL3^OE^* seedlings grown under severe Cu deficiency (MS 0 μM Cu + 100 μM BCS), Cu deficiency (MS 0 μM Cu), Cu sufficiency (MS + 1 μM Cu, control) and Cu excess (MS + 10 μM Cu). (**B**) Root length of WT (white bars) and *SPL3^OE^* (dotted bars) seedlings in the same conditions that are indicated in (**A**). The bars represent the mean ± SD of ten biological replicates. Means with a different letter are significantly different (*p* < 0.05). Scale bar = 1 cm.

To reduce the complexity of *SPL3* regulation, we determined whether the growth of the *miR156*-resistant *SPL3* overexpressing plants (*SPL3^OE^*) [16,17] would be by copper shortage. For this, WT and *SPL3^OE^* seedlings were grown for 7 d under deficiency, sufficiency and excess of copper. As shown in Figure 4, no obvious differences in growth were observed with respect to WT seedlings under Cu deficiency and sufficiency. However, under severe Cu deficiency, the root length is significantly more affected in *SPL3^OE^* seedlings (Figure 4). A slightly relative decrease in root length was also observed under Cu-excess in these seedlings (Figure 4). Aiming to exacerbate deficiency conditions, WT and *SPL3^OE^* seedlings were grown for 18 d under severe Cu deficiency (MS with 100 μM BCS) and their growth was compared to each other and to growth under Cu-sufficiency (MS with 1 μM) conditions (Figure S3A). Total root length, number of lateral roots and the growth rate, measured as the ratio of total length/number of roots, serve to quantify the observed phenotype. For all these parameters, *SPL3^OE^* seedlings show lower values than WT (Figure S3B), indicating that they are more sensitive to severe Cu deficiency.

**Figure 5:**
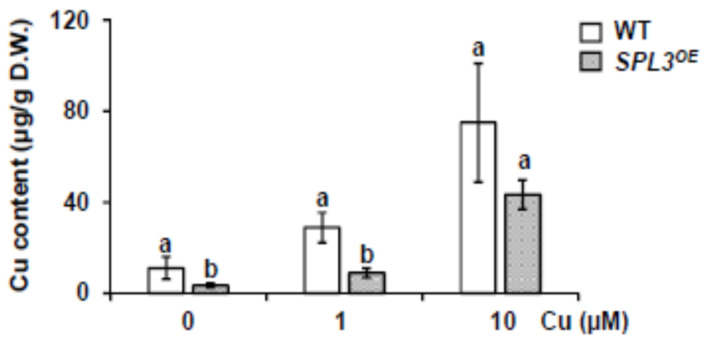
Copper content in SPL3 overexpressing plants. Total Cu content in 7-day-old WT (white bars) and *SPL3^OE^* (dotted bars) seedlings grown under Cu deficiency (MS 0 μM Cu), Cu sufficiency (MS + 1 μM Cu, control) and Cu excess (MS + 10 μM Cu). The bars represent the mean ± SD of three biological replicates. Means with a different letter are significantly different with respect to their WT (*p* < 0.05).

ICP-MS analysis revealed that copper content increased in both genotypes, in accordance with increasing copper concentrations in the medium (Figure 5). *SPL3^OE^* seedlings showed a lower copper content compared to the WT under Cu deficiency and sufficiency (Figure 5). This finding is in agreement with the observed higher sensitivity of *SPL3^OE^* seedlings to Cu deficiency.

### 2.3. SPL7-Dependent Expression in SPL3 Overexpressing Plants

**Figure 6:**
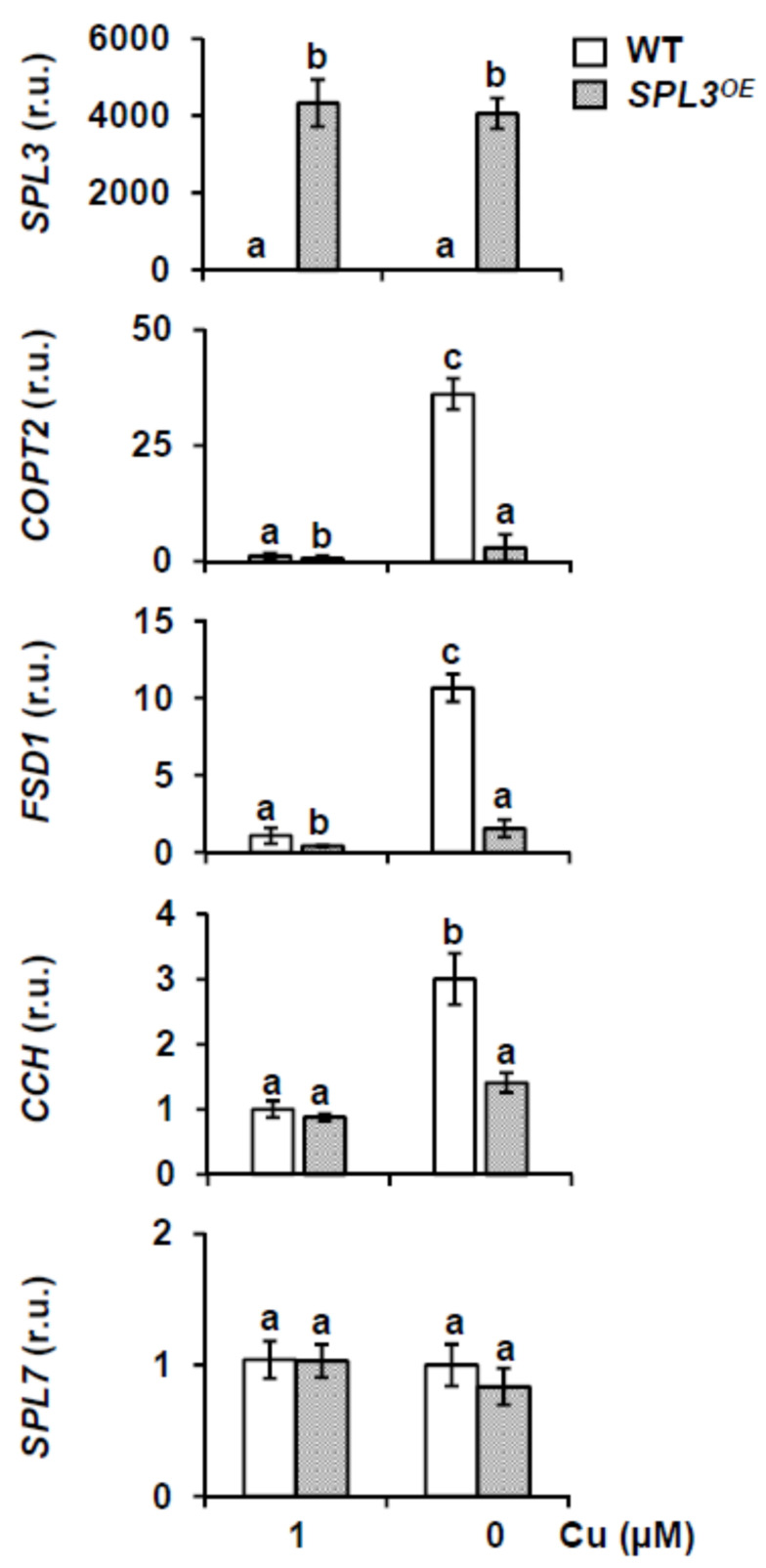
Expression of Cu deficiency markers in *SPL3^OE^* seedlings. *SPL3*, *FSD1*, *COPT2*, *CCH* and *SPL7* relative expressions in 7-day-old WT (white bars) and *SPL3^OE^* (dotted bars) seedlings grown under Cu sufficiency (MS + 1 μM Cu, control) and Cu deficiency (MS 0 μM Cu). After total RNA extraction, specific primers were used for RT-qPCR and expressed as relative units (r.u.). *UBQ10* gene was used as internal control and the WT sample grown under Cu sufficiency is arbitrarily set at one for comparison. The bars represent the mean ± SD of three biological replicates. Means with a different letter are significantly different (*p* < 0.05).

To further understand the increased sensitivity to Cu deficiency shown by *SPL3^OE^* seedlings, we first analyzed by RT-qPCR the expression of *FSD1*, *COPPER TRANSPORTER 2* (*COPT2*) and *COPPER CHAPERONE* (*CCH*), all genes typically related to Cu deficiency responses. Thereto, 7-day-old WT and *SPL3^OE^* seedlings were grown under Cu sufficiency (MS supplemented with 1 μM) and Cu deficiency (MS) medium. As a control to assess *SPL3* overexpression in our samples, the *SPL3* expression was confirmed by RT-qPCR (Figure 6). WT seedlings grown under Cu deficiency showed an increased expression of *COPT2*, *FSD1* and *CCH* with regard to the sufficiency condition (Figure 6). However, in *SPL3^OE^* seedlings this induction was about 10 times lower for *FSD1* and 12 times for *COPT2*, with respect to the induction observed in the WT. Furthermore, significant *CCH* induction under Cu deficiency conditions is not detected in the *SPL3^OE^* seedlings (Figure 6). To determine whether the attenuation of the Cu deficiency response in *SPL3^OE^* seedlings is mediated by *SPL7*, we also analyzed its expression, but found no significant differences with respect to WT seedlings, or in dependence of copper concentration in the medium (Figure 6). These results indicate that the strongly attenuated response to Cu deficiency in *SPL3^OE^* plants is not mediated by changes in the expression of *SPL7* at the transcriptional level. Furthermore, a reduced induction of the Cu deficiency response could also be observed in *SPL3^OE^* flowers, where the expression of the main SPL7-dependent targets, such as *COPT2* and *FSD1*, were again significantly reduced (Figure S5). In addition, *miR156* and *miR172* expression levels were determined in *SPL3^OE^* seedlings at different copper concentrations and they remained mostly unaffected by this (Figure S4).

To assess whether the attenuation of the Cu deficiency response observed in *SPL3^OE^* seedlings is a general effect caused by a competition for GTAC binding sites in promoters of target genes that might be caused by overexpression of any other SPL factor, we analyzed the relative expression of the Cu deficiency markers *FSD1*, *COPT2* and *CCH* in *SPL8^OE^* seedlings. *SPL8* belongs to a distinct subfamily and differs among others from *SPL3* by lacking a *miR156* responsive element [13]. As shown in Figure S6, no major changes in the relative expression of *FSD1*, *CCH* and *COPT2* genes were observed in *SPL8^OE^* seedlings with respect to WT when grown on Cu-deficient medium. These results indicate that the effects of *SPL3* overexpression on the attenuation of the Cu deficiency response are specifically mediated by SPL3 and they do not occur by overexpressing other SPL family members that are not *miR156* targets, such as *SPL8*. Although this suggests that the effects might be mediated by the *miR156-SPL* module, its universal truth remains to be investigated with other *MRE-SPL* members.

### 2.4. Cu-miRNA and Target Expression in SPL3 Overexpressing Plants

**Figure 7:**
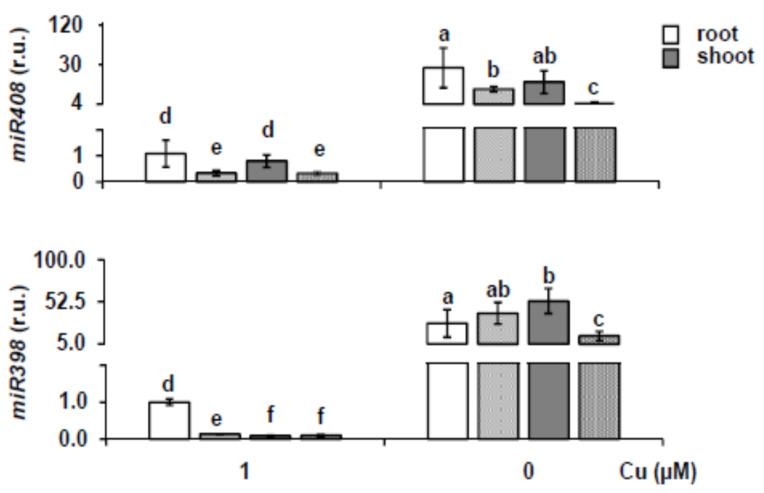
Expression of mature *miR398* and *miR408*. Relative expression of *miR398* and *miR408* genes in roots (white bars) and shoots (gray bars) from 7-day-old WT (smooth background) and *SPL3^OE^* (dotted background) seedlings grown under Cu deficiency (MS 0 μM Cu) and Cu sufficiency (MS + 1 μM Cu, control). After miRNA extraction, specific primers were used for stem-loop RT-qPCR and expressed as r.u. (relative units). *18S* gene was used as internal control and the WT root sample grown under Cu sufficiency is arbitrarily set at one for comparison. The bars represent the mean ± SD of three biological replicates. Means with a different letter are significantly different (*p* < 0.05).

In order to study the effects of *SPL3* overexpression on *Cu-miRNA* levels under Cu deficiency, the expression of two well-conserved *Cu-miRNAs*, *miR408* and *miR398*, was analyzed. As shown in Figure 7, *miR408* expression was higher under Cu deficiency, but in comparison to WT, slightly reduced in *SPL3^OE^* seedlings in both roots and shoots. This could be expected from a SPL7-mediated response and is in accordance with the previously observed attenuation of the Cu deficiency response in *SPL3^OE^* transgenics (Figure 6). Although showing a similar response to that of *miR408*, the expression of *miR398* in the roots of the *SPL3^OE^* seedlings under Cu deficiency, however, was not attenuated as expected (Figure 7).

**Figure 8:**
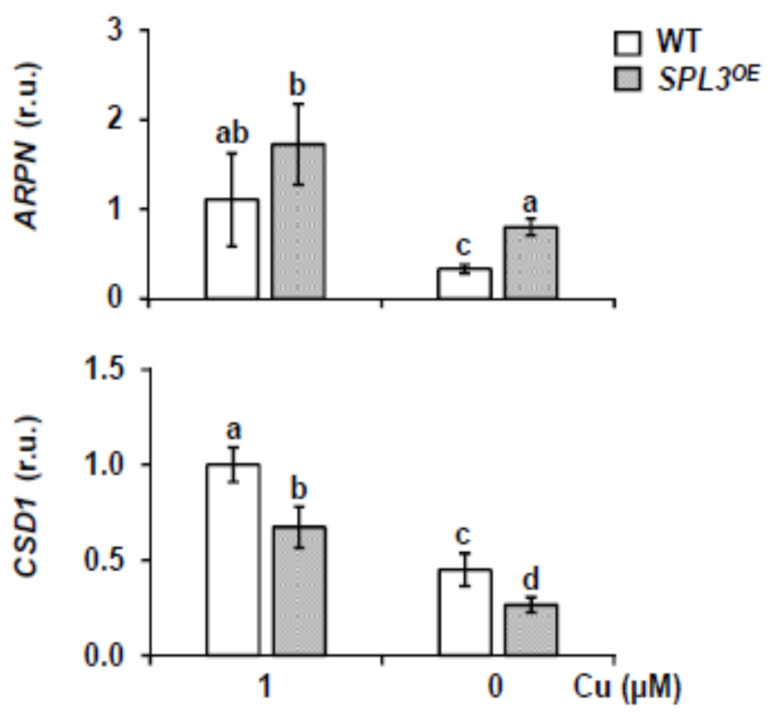
Expression of the *miR398* and *miR408* targets in *SPL3^OE^* seedlings. Relative expression of *CSD1* and *ARPN* in 7-day-old WT (white bars) and *SPL3^OE^* (dotted bars) seedlings grown in the same conditions as in Figure 5. After total RNA extraction, specific primers were used for RT-qPCR and expressed as relative units (r.u.). *UBQ10* gene was used as internal control and the WT sample grown under Cu sufficiency is arbitrarily set at one for comparison. The bars represent the mean ± SD of three biological replicates. Means with a different letter are significantly different (*p* < 0.05).

To know if their respective targets respond as expected to the changes in *miR408* and *miR398* levels as observed in the comparison between *SPL3^OE^* transgenics and WT, their expression was analyzed (Figure 8). In accordance with the relative reduced *miR408* expression, under Cu deficiency, the transcript levels of its target *ARPN* (encoding PLANTACYANIN) were found to be higher in *SPL3^OE^* compared to WT seedlings (Figure 8). However, in the case of *miR398*-targeted *CSD1* transcripts (encoding Cu/ZnSOD), the levels remained lower in *SPL3^OE^* seedlings compared to WT under any conditions (Figure 8). These results indicate that the partial down-regulation of *miR398* under Cu deficiency in *SPL3^OE^* seedlings does not translate into the induction of its target (Figure 8), possibly reflecting the interferences of other processes such as oxidative stress, in the regulation of *CSD2*.

## 3. Discussion

### 3.1. The miR156-SPL Module Affects SPL7-Mediated Cu deficiency Responses

Despite the generally observed rapid evolution of miRNAs, miR156 is highly conserved in plants as well as its function in regulating the timing of developmental transitions [63]. Moreover, Cu-miRNAs, and miR398 and miR408 in particular, are also well conserved in plants [58,59,60]. *Cu-miRNA* up-regulation under Cu deficiency is mediated by SPL7 in a relatively well-known process (SPL7/*Cu-miRNA* pathway), although other abiotic but also biotic stresses may up- or down-regulate *Cu-miRNA* expression [8,9,46,48,56]. Most small and medium size *SPLs* (*MRE-SPLs*), such as *SPL3*, are repressed by *miR156/157* at the post-transcriptional level, either through transcript cleavage or translation block. However, this last possibility has not been studied yet in detail for most *MRE-SPLs* [15,16,17]. Their larger family members (*non-MRE-SPLs*), like *SPL7*, escape from a direct miR156/157 regulation. Whereas SPL7 is considered the main regulator of the Cu deficiency response in *Arabidopsis* [9,24], to our knowledge, a role in that response for other MRE-SPL family members has not been described yet. It is therefore interesting to investigate the possibility that the *miR156-SPL* module could affect SPL7-mediated Cu deficiency responses.

**Figure 9:**
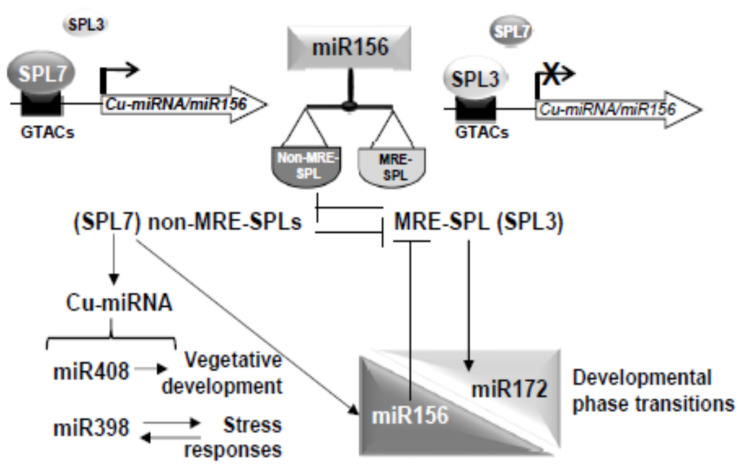
Model of the interplay between the SPL7/*Cu-miRNA* pathway and the *miR156-SPL* module. SPL7 mediated the transcriptional regulation of *Cu-miRNA*, such as *miR408* and *miR398*, and possibly the *miR156*. *miR156* expression by targeting *MRE-SPL*, such as *SPL3*, controls their transcriptional function. MRE-SPL might compete with non-MRE-SPL, such as SPL7, to bind *cis*-elements (GTACs) in the SPL target promoters precluding transcriptional productive binding. Thus, *miR156* could ultimately control the MRE-SPL/non-MRE-SPL ratio and, subsequently, their functionality. This control is exemplified by the balance between SPL3 and SPL7, as demonstrated by a Cu deficiency attenuated transcriptional response in the *SPL3^OE^* plants. The effects on *Cu-miRNA* include both common (*miR408*) and specific (*miR398*) responses depending on other environmental conditions and the degree to which the plant life cycle has progressed.

Here, we demonstrated that *miR156/157* and their target *MRE-SPL* expression is under partial control of SPL7 in *Arabidopsis* seedlings subjected to Cu deficiency. This is based on the following observations. First, *miR156/157* expression is found to be up-regulated under severe Cu deficiency and, consequently, the expression of its target *SPL3* reduced (Figure 1 and Figure 2). Second, *SPL3* expression is found to be dependent on SPL7 under Cu-excess (Figure 3A) and flowering (Figure S2). Both observations may well be explained by the direct regulation of *miR156* levels by SPL7. In agreement with this is the presence of a high number of CuRE motifs in several of the *miR156/157* promoters (Table S1—see for example the 8 GTAC in the *miR156d* promoter. Most importantly, *miR156c* up-regulation by Cu deficiency in WT shoots is almost abolished in the *spl7* mutant, as has been shown by Bernal et al. [9] in an RNA-seq experiment (WT + Cu = 0.2; WT – Cu = 0.7; *spl7* + Cu = 0.2; *spl7* – Cu = 0.3). In the same experiment, and in full accordance, the miR156 validated *MRE-SPL* targets, such *SPL2*, *SPL3* and *SPL4*, were found to be down-regulated in WT under Cu deficiency but not in the *spl7* mutant (see for example the RNA-seq values in the case of the *SPL2* target: WT + Cu = 32.1; WT – Cu = 15.7; *spl7* + Cu = 20.9; *spl7* – Cu = 20.3) [9]. Taken together, these results indicate that under Cu deficiency increased *miR156* expression, possibly *miR156c* in particular, and subsequent repression of *SPL3* (Figure 1 and Figure 2) could be mediated by SPL7 resulting in a feedback tuning of Cu deficiency responses on the *miR156-SPL* module (Figure 9).

### 3.2. SPL3 Overexpression Inhibits the SPL7 Transcriptional Activation

The other way around, the *miR156-SPL* module seems also to affect Cu deficiency responses, possibly as an integral part of developmental regulation (Figure 9). This finds support in the increased sensitivity of *SPL3^OE^* seedlings growing under Cu deficiency (Figure 4 and Figure S3) and the greatly attenuated induction of Cu deficiency markers, such as *FSD1, COPT2* and *CCH* (Figure 6) [8,9,64]. Competition between SPL7 and SPL3 TFs for GTAC binding motifs in the promoters of target genes, such as *FSD1, COPT2*, *CCH* and *Cu-miRNAs*, could provide a mechanistic explanation (Figure 9). Although this hypothesis requires further experimental confirmation, competitive binding to GTAC motifs has been proposed for small SPL members in the case of reproductive development, causing substantial functional redundancy [27]. Functional redundancy between SPL1 and SPL12 has been also reported [34,35]. Importantly, whereas SPL7 binding results in transcriptional activation of target Cu deficiency genes, the occupancy of binding sites by SPL3 seems to inhibit their transcriptional activation, possibly due to differences in the interaction with other factors. In this sense, non-productive binding by other MRE-SPLs to the GTAC motifs would redound in inhibition of the SPL7-mediated response (Figure 9). Accordingly, the *SPL3^OE^* phenotype resembled that of the *spl7* mutant with respect to the sensitivity to Cu deficiency and a lower Cu content due to the lack of *COPT2* induction [9,65]. However, relevant competition seems to be restricted to SPL3 and maybe other MRE-SPLs, since overexpression of the non-MRE-SPL, *SPL8*, has no significant effect. A plausible explanation could be that SPL8 does not contain the “extended SBP” domain, with a highly conserved sequence whose function still remains unknown [66].

Considering the well-known role of copper in plant fertility by improving grain/seed yield [1] and the established function of the *miR156-SPL* module in the control of developmental phase transitions in plants [10], next we wonder about the interplay of the *miR156-SPL* module with the SPL7-mediated Cu deficiency responses during life cycle progression. Our results suggest that both responses, the SPL7 effect on the expression of the *miR156-SPL* module (Figure 3A and Figure S2) and the *miR156-SPL* module influence on Cu deficiency (Figure 6 and Figure S5), change as the life cycle progresses. Possibly, these effects allow a stronger Cu deficiency response in seedlings compared to adult plants, since induction of Cu deficiency markers in flowers was not as strong as in seedlings (Figure 6 and Figure S5). The main phenotype of *SPL3^OE^* plants is early flowering, in accordance with SPL3 promoting the transition from vegetative to reproductive phase [67]. As copper demand is higher during reproductive development [1], SPL3 abundance may eventually interfere under Cu deficiency at that developmental stage. *SPL3* is subjected to a complex spatiotemporal regulatory network (Figure 3B). The interaction described between SPL7 and ELONGATED HYPOCOTYL5 (HY5) further underscores a connection between copper homeostasis and light [57]. Moreover, it has been described that *miR172* induces a set of *SPLs*, among which is *SPL3*, through their targets [68]. This means that *miR156* and *miR172* exert opposite effects on *SPL3* expression [22]. Although *SPL3^OE^* plants have been used here in order to circumvent the complex *SPL3* expression pattern, further research will be needed in order to sustain a physiological role of SPL3 in Cu deficiency responses at the plant reproductive phase. At the molecular level, it would be interesting to know how the SPL7 TF could be differentially functioning along the plant life cycle progression. The Cu deficiency-induced transcription factor (CIFT1) plays roles in Cu uptake into roots and copper delivery to flowers, although the interaction between SPL7 and CIFT1 in mediating Cu deficiency responses has not been completely dissected yet [69].

### 3.3. SPL7/Cu-miRNA Pathway Is Influenced by Developmental and Stress Processes

Putting the focus back on the SPL7/*Cu-miRNA* pathway, we wonder if it is globally or specifically influenced by developmental cues and by other abiotic stresses. SPL7 has been recently described to function during the adaptation to salt stress [70]. Under our experimental conditions, the expression of the *miR408* and its target, *ARPN*, is attenuated in *SPL3^OE^* plants (Figure 7 and Figure 8) in agreement with other Cu deficiency markers (Figure 6). We have recently proposed that *Cu-miRNAs* could act as post-transcriptional Modulators of Metalloprotein messenger RNA (ModMeR) [71]. Thus, *Cu-miRNAs* could participate in intracellular metal distribution by establishing a metalation ranking which prioritize essential versus non-essential metalloproteins [72]. Constitutive expression of *miR408* results in enhanced growth while decreased *miR408* expression caused impaired growth [55,56,57]. Thus, by decreasing copper delivery to extracellular multicopper oxidases, increased *miR408* levels will benefit copper delivery to essential cuproproteins, such as plastocyanin, improving plant biomass and seed yield. In this sense, *miR408* has been proposed to be a modulator of vegetative growth in response to environmental cues [55,56,57]. However, altered levels of *miR408* have detrimental effects on the response to other stresses, such as iron deficiency [54].

On the other hand, and contrary to *miR408*, the *miR398* and in particular its target, *CSD1*, showed a differential response in *SPL3^OE^* plants (Figure 7 and Figure 8) compared to other Cu deficiency markers (Figure 6). A possible explanation could be that *miR398* and/or its targets could also respond to other stress signals. Due to the role of SODs in the antioxidant responses, most probably oxidative stress signaling is part of the *miR398* expression response in *SPL3^OE^* plants. In fact, *miR398* has been reported to be up-regulated under multiple conditions where oxidative stress increased and, accordingly, putative stress-responsive *cis*-elements were found in *miR398* promoters [46]. Furthermore, *CSD2* mRNA levels were not negatively correlated with *miR398* levels under certain stress conditions, suggesting the existence of *miR398*-dependent and independent regulatory mechanisms for *CSD2* expression [46]. These results point to diverse influences on the different *Cu-miRNAs*, possibly depending on additional signaling pathways and the responsiveness of *Cu-miRNA* promoters to these signals.

Taken together, our results suggest an interaction between the *miR156-SPL* module and the SPL7-mediated Cu deficiency responses. Another putative MRE-SPL and non-MRE-SPL interaction has been also described. It has been demonstrated that *miR156*, acting through *MRE-SPLs*, such as *SPL2* and *SPL11*, is a factor involved in the tolerance to recurring environmental stress [73], whereas the *non-MRE-SPLs*, *SPL1* and *SPL12*, have been implicated in conferring thermotolerance in the reproductive stages [35]. The *miR156* levels do not only change under severe Cu deficiency and heat stress, but also in response to various other stresses such as cold, salt, and drought stress, as well as UV-B radiation, hypoxia, and biotic stress [73,74,75]. It has been hypothesized that miR156 acts as a licensing factor for the transition to flowering [22]. Based on ours and others’ results, a general picture is emerging, where the *miR156* control of the MRE-SPL/non-MRE-SPL ratio and, subsequently, their functions could be a key factor in the interplay between developmental and stress responses. Thus, stress-mediated induction of *miR156* may delay the transition to flowering by prolonging the juvenile phase and serve to avoid flowering during the stress conditions. In this way, Cu deficiency responses would be stronger in young than in adult plants, and prolonging the juvenile phase would thus help plants to overcome a period of unfavorable conditions to increase the chance of successful reproduction later. It is conceivable that the SPL7/*Cu-miRNA* pathway, by promoting vegetative growth and increased tolerance to environment abiotic stress, would be integrated with the *miR156-SPL* module in regulating these responses along the plant life cycle (Figure 9). These results reflect in part the delicate compromises that plants, being sessile organisms, need to make during different stages of their life cycle when facing nutritional deficiency and other stress conditions.

## 4. Materials and Methods

### 4.1. Plant Growth Conditions and Treatments

Seeds of *A. thaliana* ecotype Columbia-0 (Col-0), the *SPL3^OE^* and *SPL8^OE^* transgenic lines and the *spl7* mutant were surface-sterilized, stratified for 2 days at 4 °C and germinated in MS medium plates. The solution was prepared by adding macronutrients (Sigma, St. Louis, MO, USA) and micronutrients consisting of a mix of 50 mM H_3_BO_3_, 36.6 mM MnSO_4_ H_2_O, 15 mM ZnSO_4_ 7H_2_O, 0.57 mM Na_2_MoO_4_ 2H_2_O, 0.25 mM KI, and 0.05 mM CoCl_2_ 6H_2_O. Finally, 0.05% MES, 1% sucrose, and 0.8% phytoagar was added, and the pH was adjusted to 5.7–5.8 with diluted KOH. Seedlings were grown as previously described [62] for 7 or 15 days with a 12 h neutral photoperiod (65 mmol m^−2^ of cool-white fluorescent light) at 23 °C/16 °C temperature cycle. In order to obtain the indicated concentrations of Cu, the medium was supplemented with 1 or 10 µM CuSO_4_. For severe copper deficiency 100 µM BCS (Bathocuproinedisulfonic acid disodium salt) was added. Plants were grown in soil and irrigated with Hoagland’s 0.5X solution as described previously [76]. Hydroponic cultures were performed from 3–4 true leaves’ seedlings grown in commercial soil, which were transferred to black boxes containing standard Hoagland solution (0.1 X), pH 5.8, as described by [77]. After a 14-day adaptation, the Cu deficiency treatment (corresponding to a Hoagland medium without Cu sources) commenced. Media were changed weekly for 4–5 weeks. Root length was measured using the ImageJ 1.42q software (NIH Bethesda, USA) (http://rsb.info.nih.gov./ij).

### 4.2. Metal Accumulation Determinations

Cu content was determined by ICP-MS as described previously [76,78] at the Servei Central de Suport a la Investigació Experimental (SCSIE) of the Universitat de València. Briefly, lyophilized samples were digested with HNO_3_ at 100 °C and diluted with milli_Q_PLUS water (Millipore-Merck Darmstadt, Germany).

### 4.3. GUS-Staining

Assays were performed as described [79]. Briefly, the seedlings from the 7-day-old *pMIR156:GUS* and *pMIR157:GUS* transgenic lines [13] were embedded with the substrate solution (100 mM NaPO_4_, pH 7.2, 0.5 mM K_3_Fe(CN)_6_, 0.5 mM K_4_Fe(CN)_6_, 0.1% (*v*/*v*) Triton X-100, 0.5 mM 5-bromo-4-chloro-3-indolyl-β-D-glucuronide (AppliChem), and 10 mM EDTA, pH 7.2). Reactions took place at 37 °C and were stopped with ethanol (70%).

### 4.4. Gene Expression by Real-Time Quantitative PCR and Stem-Loop Quantitative PCR

Total *Arabidopsis* RNA and miRNA were isolated using the RNeasy Plant Mini Kit (Qiagen, Hilden, Germany) and MIRVANA (Ambion, Austin, TX, USA), respectively, was quantified by UV spectrophotometry and its integrity was visually assessed on ethidium bromide-stained agarose gels. After treatment with DNase I Amp Grade (Invitrogen, Waltham, MA, USA), cDNA was generated by retro-transcriptase SSII (Invitrogen, Waltham, MA, USA) as previously described [76,80]. Real-time quantitative PCR (RT-qPCR) or stem-loop RT-qPCR was carried out with SYBRGreen qPCR Super-Mix-UDG with ROX (Invitrogen, Waltham, MA, USA), with the specific primers detailed in Supplementary Tables S2 and S3, respectively, in a CFX96 Touch™ Real Time PCR Detection System (BioRad, Hercules, CA, USA), with one cycle of 95 °C for 2 min and 40 cycles consisting of 95 °C for 30 s and 60 °C for 30 s or one cycle of 95 °C for 3 min or 60 cycles consisting of 95 °C for 20 s, 53 °C for 90 s and 50 °C for 30 s, respectively. Expression values were normalized to *UBQ10* or *18S* genes, respectively, using the 2^−ddCt^ method [81].

### 4.5. Statistical Analysis

The statistical analysis of the relative gene expression was performed by the pair wise fixed reallocation randomization test (*p*-value < 0.05) [82]. For the remaining parameters, the analysis was carried out using one or two-way ANOVA with the means compared by the Duncan test (*p*-value < 0.05) using the InfoStat software, version 2010 [83].

## Data Availability

Not applicable.

## Supplementary Materials

The following are available online at https://www.mdpi.com/article/10.3390/ijms22179547/s1.
