# Supplementary material for: The Copper-microRNA Pathway Is Integrated with Developmental and Environmental Stress Responses in Arabidopsis thaliana
Source: Int J Mol Sci. 2021 Sep 2;22(17):9547. doi: 10.3390/ijms22179547 (PMC8430956; doi:10.3390/ijms22179547)
Supplement: Supplementary file 1 [file ijms-22-09547-s001.zip › ijms-1327341-supplementary.pdf]

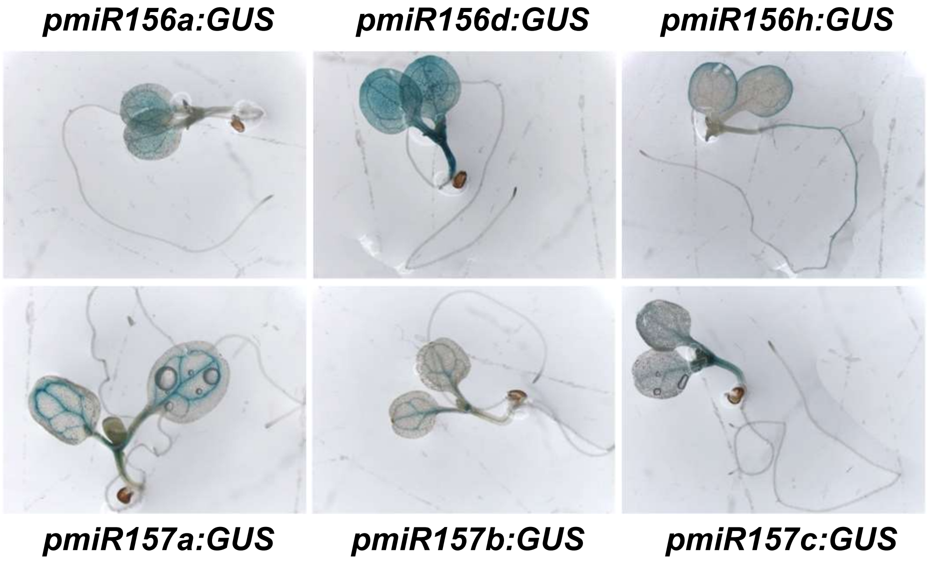

Figure S1. *miR156*- and *miR157*-driven GUS staining under Cu deficiency. GUS activity in 7-day-old seedlings of *pmiR156: GUS* and *pmiR157: GUS* transgenic lines. Overnight GUS staining in seedlings grown for under Cu deficiency (commercial ½ MS).

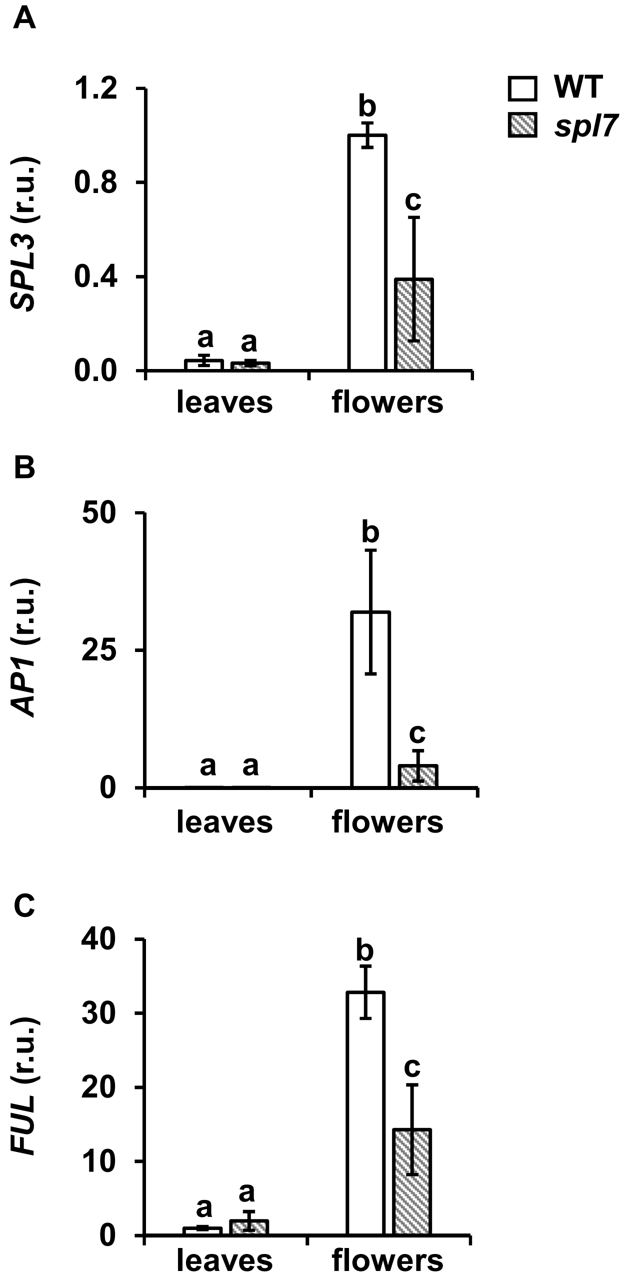

Figure S2. *SPL3* expression in adult plants. (A) *SPL3*, (B) *AP1* and (C) *FUL* relative expression of WT (white bars) and *spl7* (stripped bars) seedlings grown on soil and irrigated with Hoagland 0,5X. After total RNA extraction, specific primers were used for RT-qPCR and expressed as relative units (r.u.). *UBQ10* gene was used as internal control and the WT sample grown under Cu deficiency and WT rosette leaves are arbitrarily set at one for comparison. The bars represent the mean ± SD of three biological replicates. Means with a different letter are significantly different ($p < 0.05$).

A

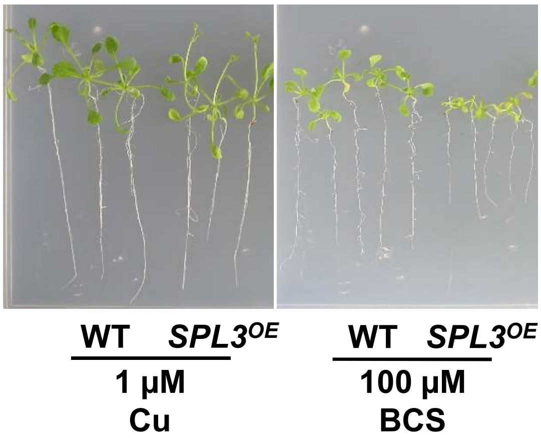

B

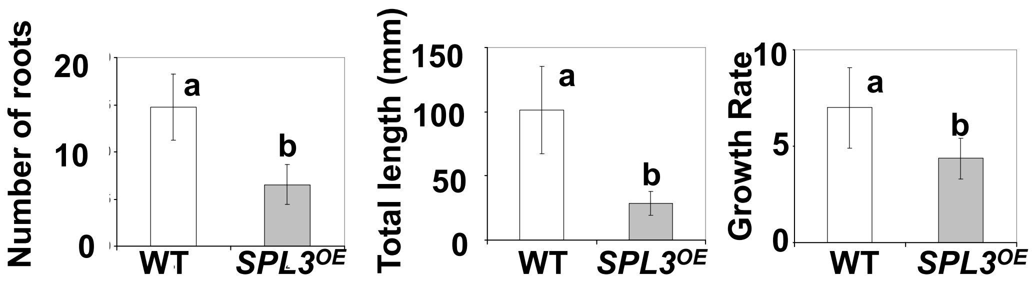

Figure S3. Phenotype of the *SPL3*^{OE} seedlings at different copper concentrations. A) Photographs of 18-day old of WT and *SPL3*^{OE} seedlings grown under severe Cu deficiency (MS 0 µM Cu + 100 µM BCS) and Cu sufficiency (MS + 1 µM Cu, control). B) Number of roots, total length and growth rate (ratio: total length/number of roots) in WT (white bars) and *SPL3*^{OE} (gray bars) seedlings under severe Cu deficiency indicated in A). The bars represent the mean ± SD of ten biological replicates. Means with a different letter are significantly different with respect to their WT ($p < 0.05$).

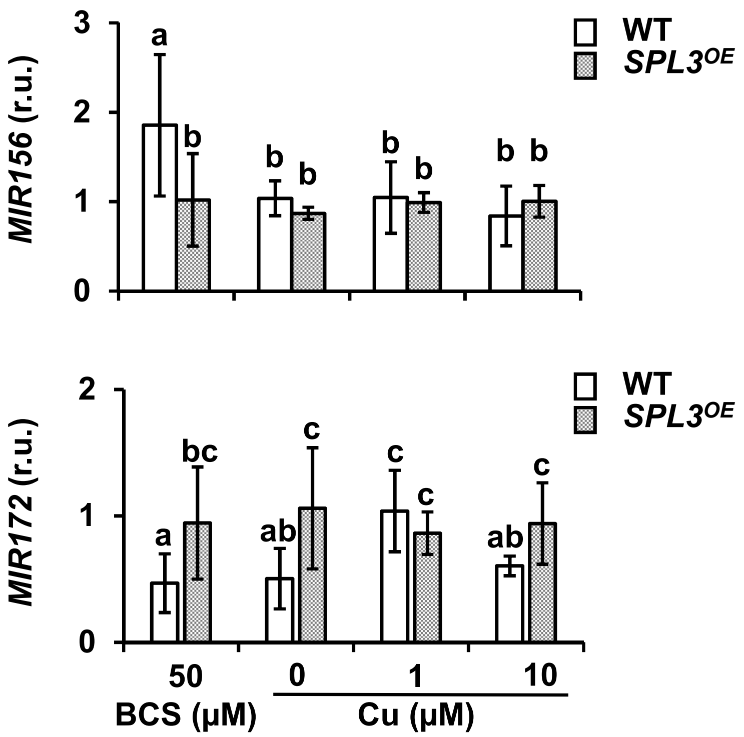

Figure S4. Expression of mature *miR156* and *miR172*. Relative expression of *miR156* and *miR172* genes in 7-day old WT and *SPL3*^{OE} seedlings grown under severe Cu deficiency (MS 0 μM Cu + 100 μM BCS), Cu deficiency (MS 0 μM Cu), Cu sufficiency (MS + 1 μM Cu, control) and Cu excess. After miRNA extraction, specific primers were used for stem-loop RT-qPCR and expressed as r.u. (relative units). *18S* gene was used as internal control and the WT sample grown under Cu sufficiency are arbitrarily set at one for comparison. The bars represent the mean ± SD of three biological replicates. Means with a different letter are significantly different ($p < 0.05$).

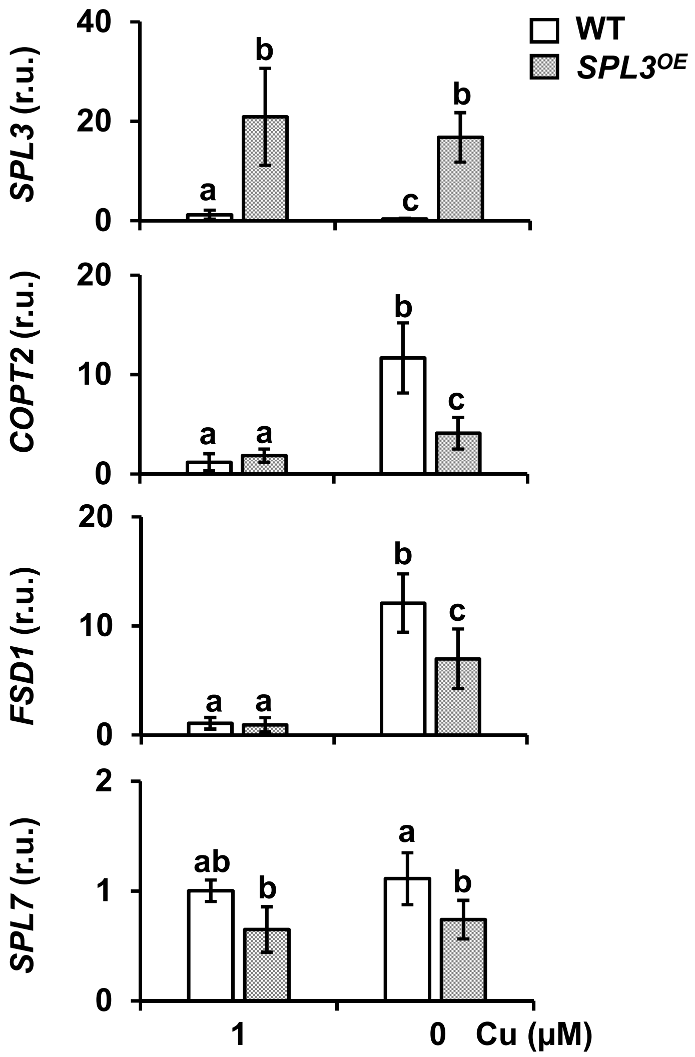

Figure S5. Expression of gene markers of Cu deficiency in *SPL3*^{OE} flowers. *SPL3*, *FSD1*, *COPT2* and *SPL7* relative expressions in 7 day-old WT (white bars) and *SPL3*^{OE} (dotted bars) seedlings grown under Cu sufficiency (Hoagland + 1 µM Cu, control) and Cu deficiency (Hoagland + 0 µM Cu). After total RNA extraction, specific primers were used for RT-qPCR and expressed as relative units (r.u.). *UBQ10* gene was used as internal control and the WT sample grown under Cu sufficiency are arbitrarily set at one for comparison. The bars represent the mean ± SD of three biological replicates. Means with a different letter are significantly different ($p < 0.05$).

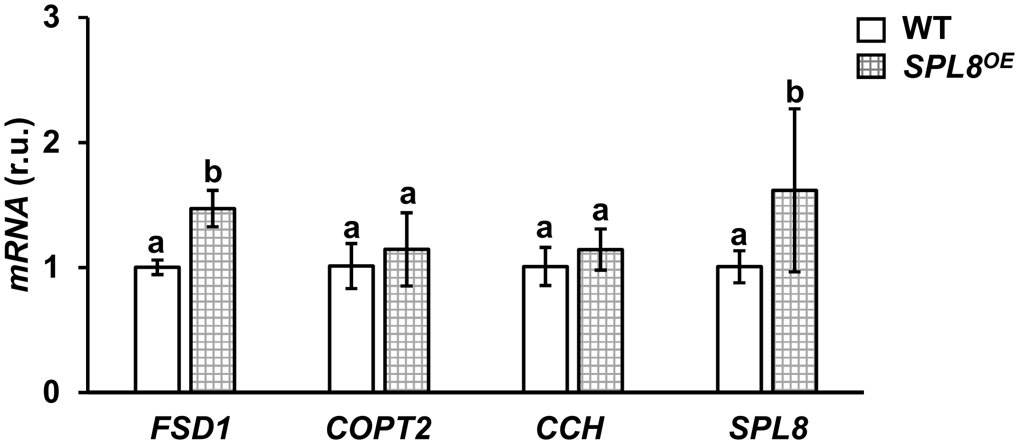

Figure S6. Expression of gene markers of Cu deficiency in *SPL8^OE^* seedlings. Relative expression of *FSD1*, *COPT2* and *CCH* in 7 day-old WT (white bars) and *SPL8^OE^* (checkered bars) seedlings grown in Cu deficiency (MS 0 μM Cu). The mRNA for each gene was analyzed by RT-qPCR with specific primers and normalized to the gene *EF1* expression. The mRNA levels are expressed in relative units (r.u.) and referred to the WT arbitrarily set at one for comparison. The bars represent the mean ± SD of three biological replicates. Means with a different letter are significantly different with respect to their WT ($p < 0.05$).

Table S1. *miR156*, *miR157* and *miR172* description. Members and MIPS and miRNA codes. GTAC elements in 2000 bp upstream of the pre-miRNA sequences.

Gene	Member	MIPS Code	miRNA Code	GTAC elements
miR156	*miR156a*	AT4G31877	MI0000178	2
	miR156b	AT4G30972	MI0000179	9
	miR156c	AT4G31877	MI0000180	1
	miR156d	AT5G10945	MI0000181	8
	miR156e	AT5G11977	MI0000182	1
	miR156f	AT5G26147	MI0000183	5
	miR156g	AT2G19425	MI0000183	4
	miR156h	AT5G55835	MI0001083	2
	miR156i	AT1G07867	MI0019232	1
	miR156j	AT2G09340	MI0019234	1
miR157	*miR157a*	AT1G66783	MI0000184	4
	miR157b	AT1G66795	MI0000185	4
	miR157c	AT3G18217	MI0000186	7
	miR157d	AT1G48742	MI0000187	12
miR172	*miR172a*	AT2G28056	MI0000215	7
	miR172b	AT5G04275	MI0000216	6
	miR172c	AT3G11435	MI0000991	9
	miR172d	AT3G55512	MI0000992	3
	miR172e	AT5G59505	MI0001089	3

Table S2. Oligonucleotides used for quantitative PCR.

**qPCR**	**Forward**	**Reverse**
AP1	AGGAGCAGTGGGATCAGCAG	TTGATACAGACCACCCATGTT
ARPN	TGACTCTCATGGCTGTGTCA	CACTACGTTGTGCATCCTCG
COPT2	CCTTTCGTATTTGGTGATGCT	AAACACCTGCGTTAAAGGAC
CCH	AAGTTGGTATGTCATGCCAA	ATATCAATGTCAAATGACTCAA
CCS	TCTCCACGTCTCTTGGGACTTT	AGCTGAGGCATGGCTCGAT
CSD1	CATCATTGGTCTCCAGGGCT	GACCTCCTTATTACATCAAT
FUL	ACGGGTCAGCAAGAAGGACA	GAGAGTTTGGTTCCGTCAACGACGATG
FSD1	ACCGAAGACCAGATTACATA	TGGCACTTACAGCTTCCCAA
LAC3	AACTGCTTTCACCAACCGTC	TGGTAGCACGAAGGACATGT
miR156a-f	TGACAGAAGAGAGT	CGCGAGCTCAGAATTAATACGA
miR157a-c	TTGACAGAAGATAGA	CGCGAGCTCAGAATTAATACGA
miR172ab	AGAATCTTGATGATGC	CGCGAGCACAGAATTAATA
miR398bc	TGGGTGTGTTCTCAGGTCA	CCAGTGCAGGGTCCGAGGT
miR408ab	TGCAATGAAAGAAGACAAAGCG	GTGCAGGGTCCGAGGT
SPL3	TGGAGTTTGTCAGGTCGAGA	AGCTAAGCGTCTCCTGCAAC
SPL7	CAGGCAGACTGTTCACCAGA	AGTTTGACGGGACCTGAATG
SPL8	TGTGCGAATTCCACTCAAAA	ATTCCGACAGCAAATGGAAC
UBQ10	TAATCCCTGATGAATAAGTGTTCTAC	AAAACGAAGCGATGATAAAGAAG

Table S3. Oligonucleotides used for Stem-loop RT.

**Stem-loop RT**	
miR156a-f miR157a-c	CGCGAGCTCAGAATTAATACGACTCACTATACGCGGTGCTC
miR172ab	CGCGAGCACAGAATTAATACGACTCACTATACGCGATGCAGCA
miR398bc	GTCGTATCCAGTGCAGGGTCCGAGGTATTCGCACTGGATACGACCAGGGG
miR408ab	CGCGAGCACAGAATTAATACGACTCACTATACGCGAGGGACCG
